# Urogenital schistosomiasis prevalence and diagnostic performance of urine filtration and urinalysis reagent strip in schoolchildren, Ethiopia

**DOI:** 10.1371/journal.pone.0271569

**Published:** 2022-07-25

**Authors:** Ketema Deribew, Delenasaw Yewhalaw, Berhanu Erko, Zeleke Mekonnen

**Affiliations:** 1 School of Medical Laboratory Sciences, Jimma University, Jimma, Ethiopia; 2 Tropical and Infectious Diseases Research Center, Jimma University, Jimma, Ethiopia; 3 Aklilu Lemma Institute of Pathobiology, Addis Ababa University, Addis Ababa, Ethiopia; Haramaya University, ETHIOPIA

## Abstract

**Background:**

Urogenital schistosomiasis has been known to be endemic in several lowland areas of Ethiopia. It is caused by *Schistosoma haematobium* and causes considerable public health problems to schoolchildren. Ethiopia, after mapping the distribution of the disease (2013 to 2015), launched school-based mass deworming program to treat schoolchildren for schistosomiasis and soil-transmitted helminthiasis (STH) across the country since 2015. However, there is no recent information about the prevalence of the disease among schoolchildren in the current study areas. Diagnostic performance of urine filtration method and urinalysis reagent strip is also lacking. Therefore, this study aimed to determine the prevalence of urogenital schistosomiasis in schoolchildren, and to evaluate diagnostic performance of urine filtration and urinalysis reagent strip in Amibara, Kurmuk and Abobo districts, Ethiopia.

**Methods:**

Across-sectional study was conducted involving 1,171 schoolchildren in Abobo, Amibara and Kurmuk districts from October, 2020 to January, 2021. The study participants were selected using random sampling technique. From each study participant, 10 ml urine samples were collected and examined using urine filtration method and urinalysis reagent strip. Data obtained from the survey were entered into Microsoft Excel 2010 and analysed with SPSS version 20.0. Data was summarized using descriptive statistics. Chi-square, bivariate and multivariable logistic regression and Pearson correlation test were used to measure associations between urogenital schistosomiasis, age, sex and haematuria. Odds ratio was used to measure strengths of association between variables. Agreement between urine filtration method and urinalysis reagent strip was determined using Kappa statistics. *P-*value < 0.05 at 95% *CI* was considered as statistically significant.

**Results:**

Among the 1,171 urine samples from schoolchildren examined by urine filtration method, 143 (12.2%) were *S*.*haematobium* egg positive. Out of 143 positive children 126(88.1%) were lightly infected and 17 (11.9%) were heavily infected. Among the total of 1,171 urine samples tested by dipstick, 264(22.5%) were positive for haematuria. Prevalence of urogenital schistosomiasis by both urine filtration and urinalysis reagent strip method was higher in Abobo than Hassoba (Amibara) and Kurmuk (*P*< 0.001). The number of egg counts (intensity of infections) were significantly correlated with intensity of haematuria (*r* = 0.6, *P* < 0.001). Egg-positive children had significantly higher risk of having haematuria compared to *S*. *haematobium* egg negative children (OR; 6.96; 95%CI: 4.98, 8.940). Compared to urine filtration method, the sensitivity, specificity, positive predictive value (PPV) and negative predictive values (NPV) of urinalysis reagent strip were 99.3%, 88.1%, 53.8% and 99.8%, respectively. Furthermore, its positive likelihood ratio (PLR) and negative likelihood ratio (NLR) were 8.34 and 0.008, respectively. The accuracy index and diagnostic odds ratio (DOR) of reagent strip were 0.89 and 1054, respectively. The agreement level between urine filtration methods and urinalysis reagent strip for detecting urogenital schistosomiasis was substantial (Kappa = 0.64).

**Conclusion:**

This study showed that urogenital schistosomiasis was prevalent in schoolchildren in Abobo, Hassoba and Kurmuk districts. Urogenital schistosomiasis prevalence in Hassoba-bure and Kurmuk falls under low category whereas moderate in Abobo and is almost four times compared to Kurmuk and Hassoba-bure. Chemotherapy is needed in schoolchildren in such endemic areas and other measures like access to safe water, improved sanitation, hygiene, and health education should be implemented to control and prevent schistosomiasis effectively. The sensitivity, specificity, positive and negative predictive values of urinalysis reagent strip were higher and could serve as alternative for mass screening of urogenital schistosomiasis, for surveillance and evaluation of schistosomiasis intervention programs.

## Introduction

Schistosomiasis is a parasitic infection and endemic in 76 countries [1, 2]. It is endemic in tropics and subtropics [3]. Intestinal and urinary (urogenital) schistosomiasis is endemic in 54 and 55 countries, respectively. [4]. Human schistosomiasis is one of the common neglected tropical diseases (NTDs) that affects around 240 million people and puts almost 800 million at risk of infection [5].

Urogenital schistosomiasis, caused by *Schistosoma haematobium* is distributed throughout Africa and the Middle East [6–9]. According to literature review and prediction, among individuals infected with *S*. *haematobium*, about 70 million experienced haematuria (blood in urine), 32 million dysuria (painful urination), 18 million bladder-wall pathology, and 10 million significant hydronephrosis within two weeks of infection in Sub-Saharan Africa [10]. This disease also causes nutritional deficiency and growth retardation [11], as well as poor cognitive development [12], decreased physical activity, poor school performance, decreased productivity and work capacity [11].

World Health Organization (WHO) had set goal to cover at least 75% of school-aged children in need of treatment against STH and schistosomiasis in all endemic countries by 2020 [12] but it did not meet the goal. Thus, WHO launched new road map for NTDs, “Ending the neglect to attain the sustainable development goals: a road map for neglected tropical diseases 2021–*2*030” which is a high-level strategic document aimed at strengthening programmatic response to NTDs and focuses on how cross-sectoral, integrated interventions, smart investment and community engagement can strengthen and sustain health systems [13].

Both *S*. *haematobium* and *S*. *mansoni* are endemic in Ethiopia, with an estimated 4 million people affected and 30 to 35 million at risk of infection [14]. *Schistosoma mansoni* is widely distributed in many parts of the country whereas *S*. *haematobium* distribution is thought to be restricted only in three lowland regions (Awash valley; Wabe-Shebele valleys and Kurmuk at the Ethio-Sudan border) [15]. Recently, *S*. *haematobium* infection is reported in Abobo town (Gambella region), Western Ethiopia [16]. Previous investigations in Afar region found *S*. *haematobium* prevalence ranging from 3.1% to 52.0% [17–19]. Geleta *et al*. [16] also reported urinary schistosomiasis prevalence in Abobo as 35.9%.

Ethiopia has successfully mapped the distribution of NTDs across the country, indicating that about 37.3 million individuals are living in schistosomiasis endemic areas [20]. Following the London Declaration on NTDs in 2012, Ethiopia’s federal ministry of health produced NTDs master plan and road map to address the country’s most prevalent NTDs [21]. Between 2013 and 2015, the prevalence of soil transmitted helminthiasis (STH) and schistosomiasis was assessed and mapped among 153,238 school-aged children (5-15yrs) across the country. The overall prevalence of schistosomiasis was 4.0%, while the prevalence of *S*. *mansoni* and *S*. *haematobium* was 3.5% and 0.3%, respectively [22]. The national mapping showed that schistosomiasis mostly occurs in West and Northeast of the country [22]. The national control program planned to achieve elimination for schistosomiasis by 2020 and aim to attain transmission interruption by 2025. Of 833 districts in Ethiopia, 374 are uninfected by schistosomiasis, 190 have low endemicity, 153 moderate endemicity, and 69 high endemicity while the rest districts not determined yet [20].

Urine filtration and urinalysis reagent strip are the most common ways of diagnosing urogenital schistosomiasis. Since the early 1980s, a reagent strip test for detection of microhaematuria has been used to diagnose *S*. *haematobium*. Some studies compared urine filtration methods to urinalysis reagent strip and concluded that detecting microhaematuria is a valid method for detecting urogenital schistosomiasis infection and related morbidity [23, 24]. A systematic review summarized that urinalysis reagent strip test for *S*. *haematobium* diagnosis has an overall sensitivity and specificity of 75% and 87%, respectively [23]. It was suggested that urinalysis reagent strip testing can be used for individual diagnosis and treatment decisions in specific situations [24, 25], however, others viewed urinalysis reagent strip testing as a valuable method for determining community prevalence in a more conservative manner [26]. In most settings, reagent strip test gives some proportion of false positive where microhaematuria could not be related with *S*. *haematobium* [27]. The prevalence of microhaematuria may not always go to zero after praziquantel treatment due to several factors such as *S*.*haematobium* egg output is generally low and thus difficult to be detected by a single filtration of 10 ml of urine, *S*. *haematobium* infections may be missed by microscopy especially in low-prevalence settings [28], the presence of bladder lesions and associated microhaematuria may last longer than the actual egg excretion [28], in females, menstrual blood or pregnancy causes reagent strip test to be positive [29]. On the other hand, different products of reagent strip work differently to detect microhaematuria, and generally, it is fair enough to say that microhaematuria is not always caused by *S*. *haematobium* infection [30, 31].

Schistosomiasis control program in Ethiopia aimed to interrupt transmission using preventive chemotherapy by 2025. Recent information on the prevalence of the disease and the diagnostic performance of urine filtration method and urinalysis reagent strip is scarce. Therefore this study aimed to determine the prevalence of urogenital schistosomiasis in schoolchildren, and evaluate diagnostic performance of urine filtration and urinalysis reagent strip in Abobo, Kurmuk and Amibara districts, Ethiopia. The findings of this study would be useful to assist public health authorities in designing and implementing proper prevention and control strategies, especially where microscopy might be hampered by factors like low intensity infection and shortage of trained human-power.

## Materials and methods

### Study area

**Fig 1:**
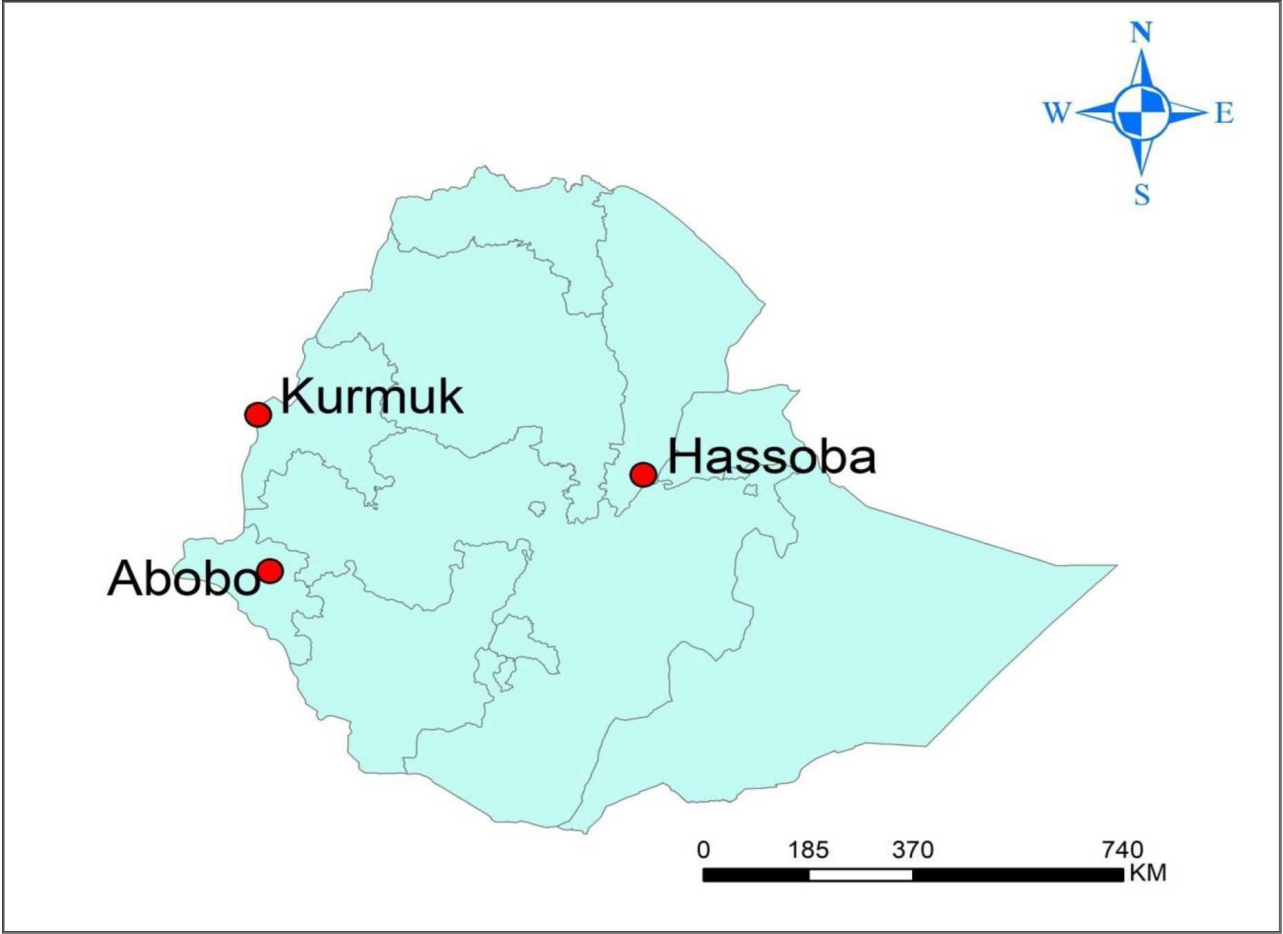
Map of study areas (source: Authors of this manuscript, created using ArcGIS version 10.5).

The study was conducted in three *S*. *haematobium* endemic areas of Ethiopia; Hassoba in Amibara district (in Afar region), Kurmuk in Kurmuk district (in Benishangul Gumuz region), and Abobo district (in Gambella region) (**Fig 1**). Hassoba village is about 290 km east of Addis Ababa with geographical coordinates 9°33’N and 40° 28’ E and an altitude of 725 m above sea level (masl). Kurmuk is about 878 Km west of Addis Ababa and near Ethio-Sudanese border. The geographical coordinates of Kurmuk district are10° 34’ N and 34° 21’ E with an altitude of 733 masl. Abobo is about 822 km Southwest of Addis Ababa with geographical coordinate of 7° 51′ N and 34° 33′ E and its altitude is about 461masl. The selected districts have limited health facilities. There is only one governmental health center in Kurmuk whereas no health centers in Hassoba-bure and Abobo villages. Overall, almost all villages in the districts are served mainly by governmental health posts.

### Study design

School-based cross-sectional study was designed to determine the prevalence of urogenital schistosomiasis and evaluate diagnostic performance of urine filtration and urinalysis reagent strip. The study was carried out among primary schoolchildren in Abobo, Amibara and Kurmuk districts from October, 2020 to January, 2021. The quantitative data were collected to assess urogenital schistosomiasis prevalence and diagnostic performance of urine filtration reagent strip in the three endemic areas of Ethiopia.

### Source and study population

The three selected districts have about 55 villages. Among these, eight villages were selected purposively for the study. From Amibara district (Hassoba and Bure villages), Kurmuk district (Kurmuk, Horzehab, Beshir, Amenzibeni) and Abobo district (Abobo and Perbongo villages5/6) were selected. These villages have a total of six primary schools with a total of 2,694 schoolchildren attending primary school during the time of data collection. From the three study areas (Hassoba, Kurmuk and Abobo), a total of **1,171** primary schoolchildren aged 5–15 years enrolled in the study since this age group is associated with highest and most stable egg out puts of schistosomiasis [32].

### Study variables

Age, gender, schistosome infection and intensity, were considered as **independent variables** whereas haematuria as **dependent variable**.

### Sample size determination

The sample size was determined for each study site using the formula:

$$n=\frac{{\left(z\right)}^{2}p(1-p)}{d^{2}}$$

and taking the none response rate of 10%, where n is the sample size, Z is 95% confidence interval (1.96), P is expected prevalence and d is precision or margin of error (5%) [33]. Previous *S*. *haematobium* prevalence was 37.0% for Hassoba [34], 50% for Kurmuk (as no recent report) and 35.9% for Abobo [16]. Based on these previous prevalence and 10% non-respondents response rate, a total of **1,171** study participants were selected from the three study areas (**387, 395** and **389** from Hassoba, Kurmuk and Abobo, respectively).

### Sampling procedure

Three districts in Gambella, Afar and Benishangul Gumuz administrative region were selected purposively since in such districts urogenital schistosomiasis is endemic. These three districts have a total of 55 villages (21 in Abobo, 19 in Amibara and 15 in Kurmuk). Among the total 55 villages in three districts, eight villages were selected purposively for this study. The study villages were selected based on their endemicity for urogenital schistosomiasis infection, and location near to dams, rivers, swamps, streams and irrigation canals. Children living in Abobo and Perbongo villages5/6 rely on Alwero dam and Alwero river water. In Hassoba and Bure village the main source of water is Awash river which used mainly for irrigation, washing clothes and bathing. Children in Kurmuk villages also depend on two major water bodies such as stream and pond water. Therefore, school-age children living in selected village with 5 to 15 years of age were eligible to be included in this study. In each village, the school student registration book was used as sampling frame and then the study participants were selected using a simple random sampling technique. During sampling gender balance was taken into consideration.

### Urine sample collection

A total of 1,171 urine samples were collected from schoolchildren after explaining the purpose of the study and obtaining informed written consent from parents/guardians and verbal assent from children. Then study participants were given a labeled plastic cup (50 ml) and asked to provide a urine sample (mid-day urine) between 10:00 am and 2:00 pm.

### Haematuria detection

On the day of collection, all urine samples (i.e. one single sample per person) were examined for microhaematuria using urinalysis reagent strip (Rapid Labs Ltd, UK). After briefly dipping the reagent strip into the urine sample (1 to 2 seconds), the strip taken out and left over the bench for about one minute. Finally, the reagent strip color was compared with corresponding reference values (color fields).The hemoglobin concentration (intensity of haematuria) in urine was recorded and graded as negative (0), trace (±), small (1+), moderate (2++) and large (3+++) according to the manufacturer’s instruction.

### Urine filtration and microscopy

Urine filtration technique was performed using polycarbonate membrane filter with 12 μm pore size (SterliTech corporation, USA), Swinnex polypropylene filter holder (SterliTech corporation, USA) and 10 ml plastic syringe. After thorough mixing, 10 ml urine was taken by syringe and pushed gently through the filter. The filter was then removed with forceps and placed on a clean glass microscope slide and examined under a 10X objective lens of a binocular microscope for schistosome eggs. The number of eggs obtained per 10 ml of urine specimen was counted and recorded. Intensity of infection was expressed as egg count per 10ml of the urine. Intensity of infection was classified as light (< 50 eggs/ 10 ml of urine) or heavy (≥ 50 eggs/10 ml of urine), according to guidelines of World Health Organization [12].

### Data analysis

Data obtained from the survey was entered into Microsoft Excel 2010 and analysed with SPSS version 20.0. Data summary was done with descriptive statistics. Comparison between the performances of urine filtration methods and urinalysis reagent strip to diagnose urinary schistosomiasis was assessed by determining sensitivity, specificity, positive and negative predictive values. Chi square, bivariate and multivariate logistic regression and Pearson correlation test were used to measure associations between urogenital schistosomiasis and haematuria. Odds ratio was used to measure strengths of association between variables. Agreement between the urine filtration method and urinalysis reagent strip was determined using Kappa statistics. Kappa value were interpreted as poor (<0.20), fair (0.21–0.40), moderate (0.41–0.60), substantial (0.61–0.80) and perfect (0.81–1.00) [35]. Statistical significance was considered at 95% confidence level (CL). *P*-value of less than 0.05 was considered significant during the analysis.

### Exclusion criteria

Schoolchildren aged below 5 years and above 15 years and female students who are onset of menstruation at the time of data collection were excluded from the study.

### Ethical consideration

Ethical approval was obtained from Institutional Review Board of Jimma University. Informed written consent was obtained from the parents/guardian of children and verbal assent from children. Briefly, orientation was given by the principal investigator and school principal in the school meeting to all parents or guardians of children in their local language and they were informed that their participation is voluntary and that they could withdraw their consent at any time and then asked to put their signature on a consent form. On the day of sample collection, only the assenting children whose consent forms were signed by their parents/guardians were enrolled. To conduct the study, permission was also obtained from the head of the district health offices as well as the principals of the schools and community leaders of the village. All the information obtained from each study participant was kept confidential. During data collection children were oriented and supported to keep physical distance in order to prevent potential COVID-19 infection. Children found positive for *S*. *haematobium* infection were treated with praziquantel at a single dose of 40 mg/kg body weight under observation of researcher and medical officers.

### Operational definitions:

**Urogenital-**relating to both the **urinary** system and the **genital** system
**Haematuria**-presence of blood in urine
**Intensity**–the quantity of schistosoma eggs found in 10ml urine sample
**Urinalysis reagent strip**-are plastic strips with pads containing chemical to test the presence of blood in urine.
**Schoolchildren**-children attending primary grades with age between 5 and15 years

## Results

### Socio-demographic characteristics of study participants

A total of 1,171 schoolchildren enrolled in this cross-sectional study. Among them, 575 (49.1%) were males (**Table 1**). The mean age (standard deviation, SD) of the study participants was 10.85 (2.78, SD) years with minimum 5 and maximum 15 years.

**Table 1:** Sex distribution of study participant’s in three study areas, Abobo, Hassoba and Kurmuk village, Ethiopia, 2021.

Gender	Study Areas			Total
	Abobo *n* (%)	Kurmuk *n* (%)	Hassoba-bure *n* (%)	
**Male**	209 (17.8%)	242 (20.7%)	124 (10.6%)	575 (49.1%)
**Female**	180 (15.4%)	153 (13.1%)	263 (22.5%)	596 (50.9%)
**Total**	389 (33.2%)	395 (33.7%	387 (33.0%)	1,171(100%)

### Urogenital schistosomiasis prevalence

As shown in **Table 2**, of the 1,171 schoolchildren, 143 (12.2% %) were positive for *S*. *haematobium* eggs. Among 143 positive school children, 77 (53.8%) were males. The dipstick test result showed that of 1,171 tested urine samples, 264 (22.5%) were haematuria positive. Prevalence of urogenital schistosomiasis infection by both urine filtration (*χ*^2^ = 7 7.97, df = 2, *P* < 0.001) and urine dipstick method (*χ*^2^ = 52.06, df = 2, *P* < 0.001) was higher in Abobo than Hassoba-bure and Kurmuk villages.

**Table 2:** Urogenital schistosomiasis prevalence by filtration method and urinalysis reagent strip test, Abobo, Hassoba and Kurmuk village, Ethiopia, 2021.

Study areas	Urine filtration test result		Reagent strip test result	
	Positive	Negative	Positive	Negative
Abobo	94	295	136	253
	(24.2%)	(75.8%)	(35.0%)	(65.0%)
Kurmuk	22	373	60	335
	(5.6%)	(94.4%)	(15.2%)	(84.8%)
Hassoba -bure	27	360	68	319
	(7.0%)	(93.0%)	(17.6%)	(82.4%)
Total	143	1028	264	907
	(12.2%)	(87.8%)	(22.5%)	(77.5%)

**Fig 2:**
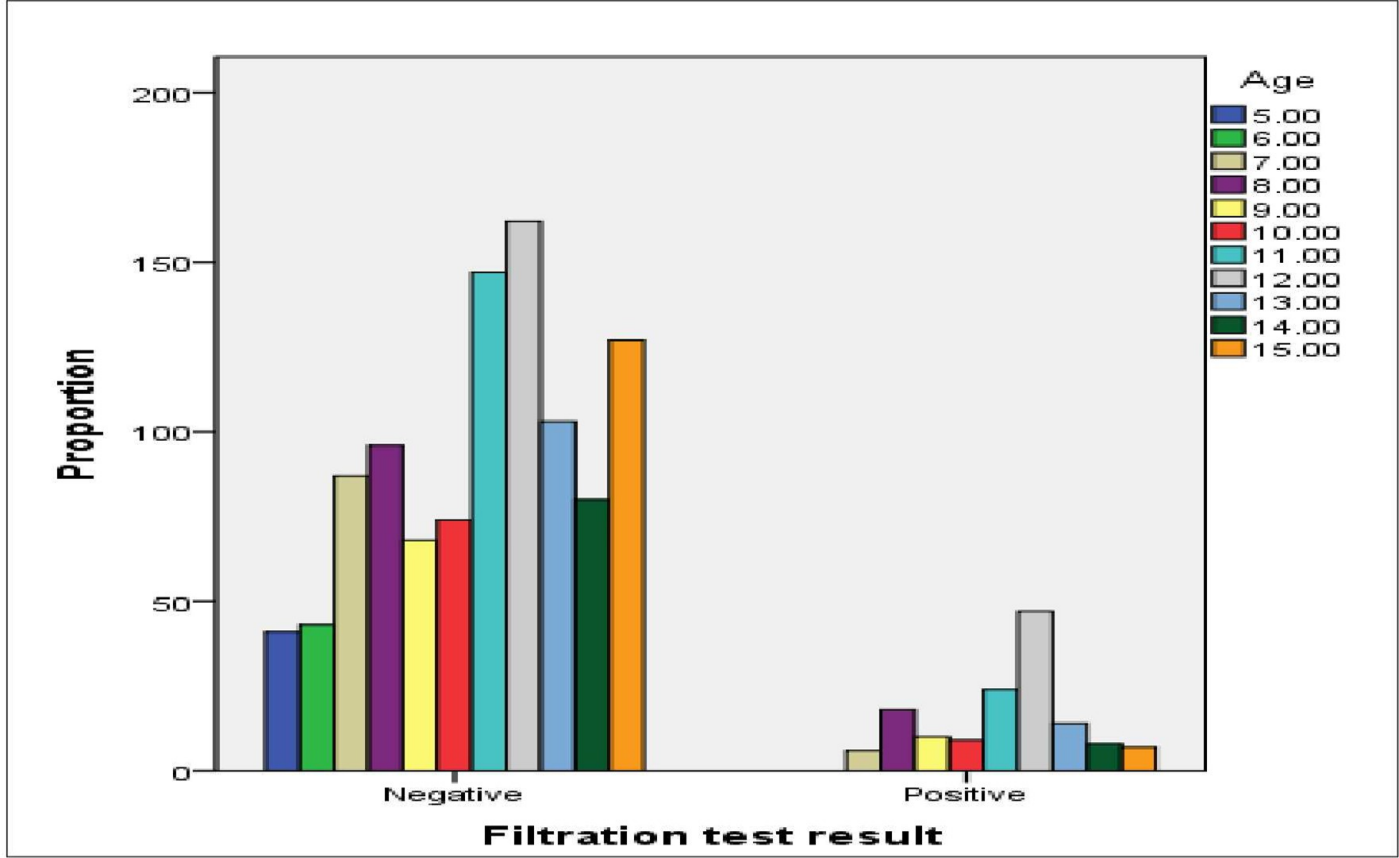
Prevalence of urogenital schistosomiasis by age (using urine filtration method).

The prevalence of urogenital schistosomiasis was not associated with gender (*χ*^2^ = 1.46, df = 2, *P* = 0.23) but significantly associated with age (*χ*^2^ = 44.12, df = 10, *P* < 0.001). The Highest prevalence of schistosomiasis was observed in schoolchildren with age of 12 years (**Fig 2**).

### Urogenital schistosomiasis intensity

The mean (SD) egg count was 2.5 (9.8) with minimum of two eggs and maximum 95 eggs per 10ml urine. Of 143 positive children, 126(88.1%) were lightly infected (< 50 egg counts/10 ml of urine) and 17 (11.9%) were heavily infected (≥ 50 egg counts/10 ml of urine). Heavy infection intensity in females was higher than males but the difference was not statistically significant (*p* = 0.06).

### Association between *S*.*haematobium* infection and haematuria

The majority of urine samples were lightly colored and only very few children’s urine 71 (6%) had visible haematuria (macrohaematuria). Among the 143 *S*.*haematobium* egg-positive urinesamples, 142(99.3%) were microhaematuria positive and among the 1028 *S*.*haematobium* egg negative urine samples, 122(11.8%) were microhaematuria positive (**Table 3**).There was positive correlation (r = 0.69, *P* = 0.01) between infection intensity and haematuria intensity (grade levels). Compared with *S*. *haematobium* egg negative urine samples, egg positive urine samples had significant association with microhaematuria (*χ*^2^ = 1069.1, df = 10, *P* = 0.001). Bivariate regression analysis showed that egg-positive children had significantly higher risk of having haematuria compared to *S*.*haematobium* egg negative children (COR; 6.96; 95%CI: 4.98, 8.940). Age and sex adjusted multivariable logistic regression analysis also showed higher risk of haematuria in children who are positive of urogenital schistosomiasis (AOR; 6.95; 95%CI: 4.96, 8.92).

**Table 3:** Association between *S*.*haematobium* infection and haematuria in schoolchildren, in the three study area (Abobo, Hassoba and Kurmuk village), Ethiopia, 2021.

***Schistosoma haematobium* egg**	**Haematuria**			**COR (95%CI)**	**AOR (95%CI)**
	**Positive**	142	1	6.96 (4.98,8.94)	6.95 (4.96,8.92)
	**Negative**	122	906	1	1

**AOR-** adjusted odd ratio; **CI-** confidence interval; **COR-** crude odd ratio

### Sensitivity and specificity of the Urinalysis reagent strip

Urine filtration method was used as a reference to evaluate the diagnostic performance of urinalysis reagent strip. Of the total 1,171 samples, 142 (12.12%) were positive by both urine filtration method and urinalysis reagent strip, while 906 (77.36%) were found negative by both methods as shown in **Table 4**. When compared with urine filtration method, the sensitivity, specificity, positive predictive value (PPV) and negative predictive values (NPV) of urinalysis reagent strip were calculated to be 99.3%, 88.1%, 53.8% and 99.8%, respectively. Positively likelihood ratio (PLR) and negatively likelihood ratio (NLR) of the reagent strip are calculated as 8.34 and 0.008. Only one urine sample test was negative by urinalysis reagent strip test but positive by urine filtration method. The accuracy index and diagnostic odds ratio (DOR) of reagent strip were 0.89 and 1054, respectively. The level of agreement between the two diagnostic methods for detecting the presence of urogenital schistosomiasis was substantial (Kappa = 0.64).

**Table 4:** Diagnostic performance of urinalysis reagent strip compared with urine filtration for the diagnosis of *S*. *haematobium* in school children, Abobo, Hassoba and Kurmuk village, Ethiopia, 2021.

		Urine Filtration test result		Total
		Positive	Negative	
**Urinalysis reagent strip test result**	**Positive**	142(TP)	122(FP)	264
	**Negative**	1(FN)	906(TN)	907
**Total**		143	1028	1171

**TP** true positive, **TN** true negative, **FP** false positive, **FN** false negative

## Discussion

This study was conducted to determine the diagnostic performance of urine filtration method and urinalysis reagent strip and to assess the prevalence of urogenital schistosomiasis in schoolchildren living in Hassoba-bure, Kurmuk and Abobo villages, Ethiopia. The overall prevalence of urogenital schistosomiasis was 12.2% in three endemic areas. The prevalence of urogenital schistosomiasis among children in Hassoba-buri, Kurmuk, and Abobo villages was 7.0%, 5.6%, and 24.2%, respectively. Higher proportion of urogenital schistosomiasis was observed in Abobo district than Kurmuk and Hassoba-bure. Previous study in Hassoba-bure documented a prevalence of 24.5% for *S*. *haematobium* infection in schoolchildren [17]. Similarly, Degarege *et al*. [34] also reported prevalence of urogenital schistosomiasis in Hassoba and Buri village as 37% and 25%, respectively. Geleta *et al*. [16] also reported urinary schistosomiasis prevalence in Abobo as 35.9%. However, prevalence of urinary schistosomiasis in Abobo in the present study reported as 24.2%. Birrie *et al*. [36] had reported a decline of urogenital schistosomiasis from 30.2% (1981) to 5.7% in 1996 in Kurmuk but our study showed a prevalence of 5.6% with no decline over such a longer period of time.

The drop in urogenital schistosomiasis prevalence in Hassoba-bure and Kurmuk could be attributed to the drying out of most marshes and ponds (which negatively affects intermediate snail hosts of *S*.*haematobium)*, and ongoing mass drug administration (MDA) targeting schoolchildren. Over 19 million people have been treated for schistosomiasis and STHs since the nationwide control program began in November 2015. Furthermore, in some village’s recent safe water supply may have contributed to a reduction in cercariae-infested rivers, streams, ponds and dam water contact activities. Urogenital schistosomiasis showing decline in general but in Abobo the prevalence is greater compared to Hassoba-bure and Kurmuk. This may be due to the fact that in Abobo water contact activity at Alwero dam was higher indicating probable re-infections.

This study showed urogenital schistosomiasis infection rate variation with age of children. The disease was highly associated with age 11 & 12 years and the highest infection was recorded at age of 12 years. Similar studies reported that prevalence of urogenital schistosomiasis was higher among children aged above 10 years compared to those aged below 10 years [37]. In other study, the highest number of positive cases was reported among children whose age is 12 to 15 years [38]. Senghor *et al*. [39], recorded the highest prevalence values of urogenital schistosomiasis in children with age group of 10–15 years. Increased infection rate of urogenital schistosomiasis in children was also reported in Darfur (Sudan) in children aged 10–14 years [40]. Geleta *et al*. [16] also reported the higher infection rate of urogenital schistosomiasis in age group 13 to 14 years than 7 to 9 years in Abobo (Ethiopia).This may be due to the fact that children in this age group have excessive mobility and they may become more exposed to cercariae infested water while swimming/playing, fetching water and agriculture activities.

Prevalence of urogenital schistosomiasis was 12.2% using urine filtration method but prevalence increased to 22.5% when the same urine samples were diagnosed with urinalysis reagent strip. Several reasons such as detection of *S*. *haematobium* infection using filtration method may miss cases especially during light infection and the true prevalence might be underestimated [41, 42]. In this study, the majority (88.1%) of *S*. *haematobium* infection cases had a light intensity of infection, and the reagent strip detects the majority (99.4%) of cases as positive. In most settings, reagent strip test results in some proportion of false positive where microhaematuria could not be related with *S*. *haematobium* [27].

In this study female participants who were on menstrual cycle at the time of data collection were informed not to deliver urine sample since the urinalysis reagent strip method affected by menstrual blood and other genitourinary infections [43]. However, this was not entirely feasible to rule out, and it may have contributed to the increased reporting of the parasite’s prevalence in some locations or locales. Indeed, the prevalence of haematuria in children was higher in males than females in the current study, although the difference was not statistically significant. Similarly, study in Nigeria found no significant differences in the prevalence of microhaematuria based on age or gender [44].This is due to the fact that blood in urine is a characteristic sign of urogenital schistosomiasis in endemic communities where its prevalence correlated positively with urinary schistosomiasis [45].

This study showed no significant association between gender and urogenital schistosomiasis but relatively males are more infected than females. This is in agreement with other studies showing no difference between male and female [34, 46–48]. This may be due to the fact that both male and female children are engaged equally in activities like fetching water, washing clothes, bathing and other activities that expose them to cercariae-infested water.

Urinalysis reagent strip test in this study showed higher positive test results (22.5%) compared to urine filtration (12.2%). Similar study in Nigeria showed higher positive test results by urinalysis reagent strip test than urine filtration [49]. Detecting schistosome eggs in urine using a filtration method is a reliable way for diagnosing urogenital schistosomiasis. In this study, the sensitivity and specificity of urinalysis reagent strip with reference to urine filtration method were 99.3% and 88.1%, respectively. This study showed higher sensitivity and specificity of the urinalysis reagent strips. This finding is in consistent with earlier studies in various schistosomiasis transmission settings in sub-Saharan Africa (SSA) [23, 26, 50–52]. The specificity and NPV were high, similar to previous studies [23, 24]. However, in other low transmission setting, reagent strips have been observed to have a low specificity [23]. In the low transmission area, the PPV was quite high, which increases the chances of identifying people who had false negative results. This outcome is consistent with findings from prior investigations in low-transmission settings [26, 53]. The high PPV of urinalysis reagent strip, on the other hand, is comparable to other SSA areas with moderate transmission [26, 50, 54]. As a result of these differences in urinalysis reagent strip performance in low transmission settings, positive results must be confirmed using more sensitive diagnostic methods.

The strength of our study is that data collection was done under close follow up and supervision of authors. Furthermore, few urine samples (40 samples per day) was collected and analysed to determine intensity of infection effectively and efficiently. To get reliable test results we employed urinalysis reagent strip which was not opened and stored in a correct way as per the manufacturer’s instruction.

This survey is one of the few studies conducted in Ethiopia on the epidemiology of urogenital schistosomiasis in schoolchildren and diagnostic performance of urine filtration method and urinalysis reagent strip. The country has a national deworming program targeting school age children. Therefore, finding of this study would be helpful as it showed the risk of urogenital schistosomiasis in schoolchildren. Furthermore it showed the diagnostic performance of urine filtration method and urinalysis reagent strip which is a common diagnostic approach in epidemiological studies. This study also showed how schistosomiasis control program such as MDA is effective in schoolchildren in the study areas.

One of the limitations of this study is the use of a single urine sample for assessing the diagnostic performance of urine filtration methods and urinalysis reagent strip. Since the multiple urine samples from the same individuals would have allowed assessing more thoroughly the association between urogenital schistosomiasis and haematuria while accounting for variation between individuals as well as changes in egg output during the day. The amount of *S*.*haematobium* egg excreted in urine may vary with days [55]. Having samples collected on several days, the chance of missing light infections could be less. Thus, the prevalence of *S*.*haematobium* infection in the current study areas might be underestimated by filtration method. Some information on socio-economic characteristics, water contact patterns, and other risky practices related to schistosomiasis transmission are not included in this study so further study should consider these surveys. Finally in this study highly sensitive diagnostic method such as PCR was not used to validate urine filtration and reagent strip methods.

## Conclusion

Urogenital schistosomiasis was prevalent in three urogenital schistosomiasis endemic areas of Ethiopia. Schistosomiasis prevalence in Hassoba-bure and Kurmuk can be categorized as low-risk but moderate-risk in Abobo according to WHO recommended treatment strategy for schistosomiasis [56]). Therefore, school children in Kurmuk and Hassoba needed to be treated once every three years whereas once in every two years in Abobo according to WHO recommendations. Urinalysis reagent strip was highly sensitive and specific. It can be considered as reliable diagnostic tool for the rapid diagnosis of urinary schistosomiasis in a low-resource, in highly endemic context because of its accuracy, ease of use, and quick interpretation. So, it can be used for surveillance and evaluation of schistosomiasis intervention program. In addition to treatment with praziquzntel, schoolchildren living in endemic villages of the Kurmuk, Abobo and Hassoba-bure village should have access to safe water, improved sanitation, hygiene, and health education to prevent and control schistosomiasis.
